# Maternal depressive symptoms in childhood and risky behaviours in early adolescence

**DOI:** 10.1007/s00787-017-1043-6

**Published:** 2017-09-13

**Authors:** Eirini Flouri, Sofia Ioakeimidi

**Affiliations:** Department of Psychology and Human Development, UCL Institute of Education, University College London, 25 Woburn Square, London, WC1H 0AA UK

**Keywords:** Attitudes to alcohol, Maternal depression, Moral judgement, Youth risky behaviours

## Abstract

Longitudinal patterns of maternal depressive symptoms have yet to be linked to risky behaviours, such as substance use or violence, in early adolescence, when such behaviours may be particularly detrimental. This study was carried out to do this. Using data from the UK’s Millennium Cohort Study, it modelled the effect of trajectories of maternal depressive symptoms at child ages 3, 5, 7 and 11 years on antisocial behaviour and delinquency at age 11 years (*N* = 12,494). It also explored their role in predicting moral judgement and attitudes to alcohol at age 11, important predictors of delinquent or antisocial behaviour and alcohol use, respectively. Latent class analysis showed four longitudinal types of maternal depressive symptoms (chronically high, consistently low, moderate-accelerating and moderate-decelerating). Maternal symptom typology predicted antisocial behaviour in males and attitudes to alcohol in females, even after adjusting for youth’s age and pubertal status and after correcting for confounding. Specifically, compared to males growing up with never-depressed mothers, those exposed to chronically high or accelerating maternal depressive symptoms were more likely to report engaging in loud and rowdy behaviour, alcohol use and bullying. Females exposed to chronically high maternal depressive symptoms were more likely than those growing up with never-depressed mothers to support the view that alcohol use is harmless. While causal conclusions cannot be drawn, these findings suggest that preventing or treating maternal depressive symptoms in childhood may be a useful approach to reducing future externalising and health-risk behaviours in offspring.

## Introduction

Previous research has demonstrated strong effects of longitudinal patterns of maternal depression on adolescent internalising and externalising problems [13–15, 17]. Associated with both maternal depression and internalising/externalising problems [18, 22] are risky behaviours, such as substance use, truancy and delinquent or antisocial behaviour. To the best of our knowledge, however, only two studies have investigated the role of longitudinal patterns of maternal depression in youth risky behaviours [1, 16]. These studies established that youth exposed to high levels of maternal depressive symptoms throughout childhood engage in more risky behaviours than their counterparts. However, in both these studies the risky behaviours explored were in late or middle adolescence (16–17 and 15 years, respectively). We do not know the association between trajectory types of maternal depressive symptoms and offspring risky behaviours in early adolescence, when such behaviours may be detrimental for later outcomes [9, 10, 23–25].

We carried out this study to address this gap. Using a large population-based longitudinal sample from the UK, the Millennium Cohort Study (MCS), we investigated the role of trajectory types of maternal depressive symptoms at child ages 3–11 years in risky behaviours, such as delinquency and alcohol use, at age 11 years. We also explored their role in attitudes towards a range of antisocial activities and alcohol, strong predictors of delinquency and alcohol use in youth, respectively [3, 4]. We examined all these relationships separately for males and females, in view of the sex differences in youth risky behaviours [1–4].

### The present study

We met our study aim in three steps. First, we specified the constructs of risky behaviours by the self-reported items indexing the broad cluster of risky behaviours in MCS at age 11 years. Next, we described the trajectories of maternal depressive symptoms, measured at child ages 3–11 years. Finally, we examined if the trajectories were related to youth risky behaviours even after adjusting for important demographic and socio-economic factors related to both. These factors were family poverty, maternal education and age, ethnicity, and number of siblings and family disruptions [5]. All models also controlled for pubertal status, given that earlier and faster maturation predicts delinquency and other risky behaviours in adolescence [6] and is related to maternal depression [28].

## Method

### Participants and procedure

MCS is a population-based longitudinal cohort of children born in the UK over 12 months from 1 September 2000. Children were around 9 months old at Sweep 1, and around 3, 5, 7 and 11 years old at Sweeps 2, 3, 4 and 5, respectively. MCS was designed to over-represent families living in areas of high child poverty, areas with high proportions of ethnic minority populations across England, and the three smaller UK countries. Parent-reported data were collected through interviews and self-completion questionnaires. Ethical approval was gained from NHS Multi-Centre Ethics Committees, and parents gave informed consent before interviews took place. At Sweep 1, 18,522 families participated in MCS. The numbers of productive families at Sweeps 2, 3, 4 and 5 were 15,590, 15,246, 13,857 and 13,287, respectively. In all, 19,244 families participated in MCS. The analytic sample was children (singletons and first-born twins or triplets) whose mothers had valid data on depressive symptoms in at least one of Sweeps 2–5 and with at least one measure of risky behaviours at age 11 years (*N* = 12,494). We excluded Sweep 1 as parental depressive symptoms were measured differently at the beginning of MCS.

### Measures

Youth risky behaviours were measured at age 11 years by asking cohort members questions about their engagement in various risky behaviours (e.g., use of alcohol or cigarettes, truancy, and delinquent and antisocial behaviours such as stealing or destroying others’ property) and bullying. The questionnaire used was adapted from the age 3 survey of the cohort members’ older siblings (not part of MCS). The questionnaire also attempted to measure moral judgement (by attitudes to illegal or antisocial activities such as stealing, fighting or destroying others’ property) as well as attitudes to alcohol. Maternal depressive symptoms were measured at child ages 3, 5, 7 and 11 years with the Kessler K6, a 6-item screener of psychological distress with robust psychometric qualities [7]. The family and maternal covariates examined were maternal age at the beginning of the study period (Sweep 2), ethnicity (white, black, Indian, Pakistani/Bangladeshi, mixed and other), number of siblings and maternal education (university degree or not) by the end of the study period (Sweep 5), number of family disruptions (family status changes) throughout the study period (Sweeps 2–5), and number of sweeps in poverty (below the poverty line) throughout the study period. Youth’s pubertal status (some signs of puberty vs. no signs) was measured with the parent’s report at Sweep 5 of whether or not there was breast growth or menstruation or hair on body (for females), and voice change or facial hair or hair on body (for males). In our sample, 40% of males and 85% of females had shown at least some signs of puberty.

### Analytic approach

To explore if maternal depressive symptoms followed distinct trajectories, we fitted latent class models in Mplus. In latent class analysis, longitudinal trajectories are unknown but can be inferred from patterns of responses on observed indicators (in this case, maternal depressive symptoms) measured over time. Latent class analysis can summarise these patterns by creating longitudinal profiles in a parsimonious way. We used the full information maximum likelihood method which is naturally incorporated into the generalised latent variable modelling framework to estimate parameters and standard errors from the available data, under the assumption that missingness is at random given the variables in the model and that the models are correctly specified. To determine whether the identified maternal trajectories predicted youth outcomes even after accounting for possible confounders, youth risky behaviours at 11 years were regressed on the trajectory membership variables and the covariates (maternal age and education, ethnicity, family poverty, number of siblings and family disruptions, and pubertal status) in linear regression models using SPSS. Attrition/non-response and survey design were taken into account by using weights in all analyses. The linear regression analysis was performed using the Complex Samples General Linear Model procedure to account for this weighting. As explained, all regression models were stratified by sex.

## Results

Table 1 shows the prevalence rates for each of the categorical outcomes (engagement or no engagement in risky behaviours) and the means and standard deviations for each of the continuous variables, separately for males and females. As can be seen, a higher proportion of males than females engaged in risky behaviours. Compared to females, males also reported lower moral judgement and, in general, more positive attitudes to alcohol.

**Table 1:** Risky Behaviours and Attitudes by Sex

Items	Males		Females		*p*
	Categorical variables				
	*N*	%	*N*	%	
Have missed school without permission	269	5.0	140	2.6	<0.001
Have bullied other children	2085	35.5	1364	23.8	<0.001
Have tried a cigarette	213	3.9	119	2.3	<0.001
Have had an alcoholic drink	882	15.6	571	11.0	<0.001
Have been noisy or rude in public	1420	23.9	794	13.6	<0.001
Have shoplifted	380	7.0	241	4.3	<0.001
Have sprayed graffiti	223	3.7	133	2.1	<0.001
Have damaged others’ property	236	4.4	68	1.2	<0.001

	Continuous variables				
Items (range)	*N*	Mean (SD)	*N*	Mean (SD)	*p*
Starting a fight not very wrong (1–3)	5867	1.43 (0.01)	5856	1.28 (0.01)	<0.001
Spraying graffiti not very wrong (1–3)	6068	1.10 (0.00)	6094	1.06 (0.00)	<0.001
Shoplifting not very wrong (1–3)	6109	1.07 (0.00)	6137	1.04 (0.00)	<0.001
Copying/downloading music/games/films illegally not very wrong (1-3)	5898	1.33 (0.01)	5925	1.21 (0.01)	<0.001
Drinking is a way to make friends (1–4)	6093	1.52 (0.01)	6128	1.40 (0.01)	<0.001
Drinking makes people worry less (1–4)	5972	2.02 (0.01)	6016	1.85 (0.01)	<0.001
It is easier to open up after a few drinks (1–4)	5787	2.02 (0.01)	5816	1.92 (0.01)	<0.001
Drinking alcohol makes people happier with themselves (1–4)	5856	1.99 (0.01)	5911	1.82 (0.01)	<0.001
Drinking alcohol does not get in the way of school work (1–4)	5892	1.81 (0.01)	5955	1.82 (0.02)	0.60
Drinking alcohol does not make it hard to get along with friends (1–4)	5811	2.15 (0.02)	5815	2.18 (0.01)	0.12
If I drank alcohol without my parents’ permission, I would not be caught and punished (1–4)	5957	1.48 (0.01)	5986	1.47 (0.01)	0.39

Ns are unweighted; means and percentages are weighted

To decide on the risky behaviours for the regression models, we carried out three principal components analyses (PCAs) with oblimin rotation. The first PCA was carried out on the dichotomous items measuring cigarette use, alcohol use, bullying, truancy and the four criminal and antisocial behaviours (shoplifting, spraying graffiti, property damage, and loud and rowdy behaviour). The second PCA was carried out on the four Likert-scale items measuring moral judgement. The third PCA was carried out on the seven Likert-scale items measuring attitudes to alcohol. The first PCA revealed two factors, ‘delinquency’ (loading on truancy, spraying graffiti, cigarette use, shoplifting and property damage) and ‘antisocial behaviour’ (loading on loud and rowdy behaviour, bullying and alcohol use). The second PCA showed one factor, ‘low moral judgement’. The third PCA revealed two factors. The first factor, ‘positive attitudes to alcohol’, was indexed by the four items supporting the views that alcohol has social benefits and mood-enhancing properties. The second, broadly described as ‘low perceived harm of alcohol use’, was indexed by the three items opposing the views that alcohol use interferes with school work, damages personal relationships and is disapproved of by parents.

**Fig. 1:**
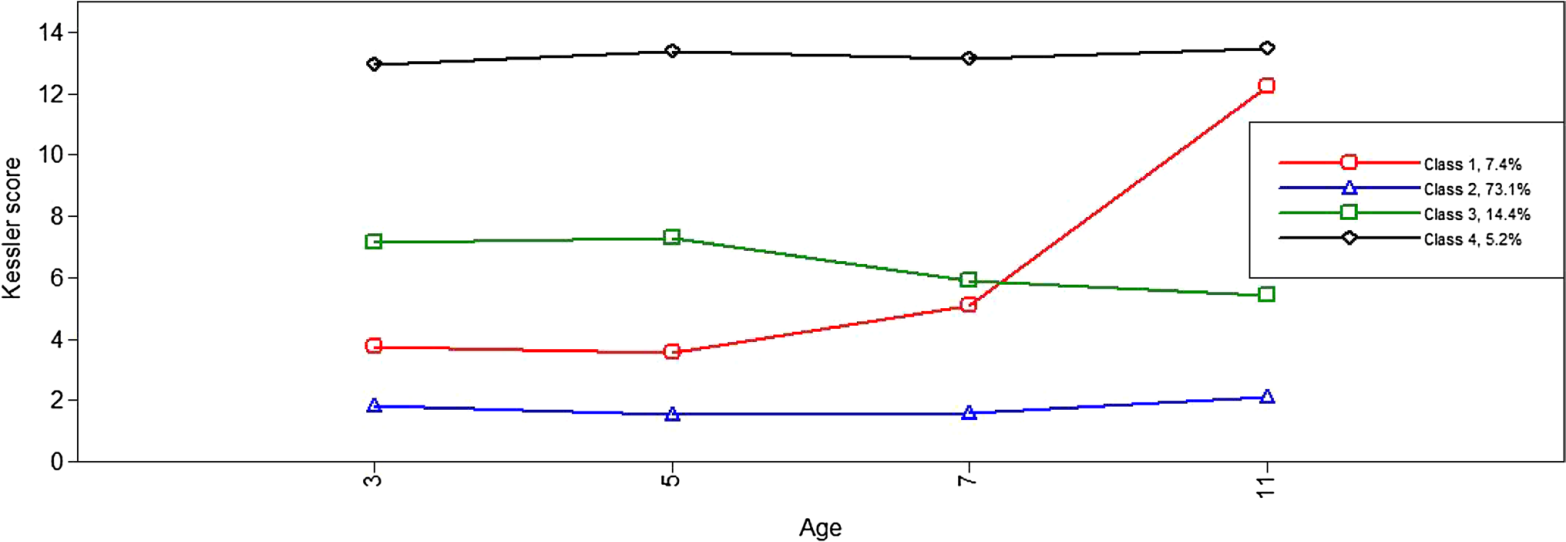
The four-class solution of maternal depressive symptoms (*Y* axis) at child ages 3, 5, 7 and 11 years (*X* axis)

In Table 2 we present information criteria, log-likelihoods and the entropy coefficient, a measure of classification quality (values close to 1 indicate good allocation quality and low classification error) for different specifications of the latent class model. As expected, model fit improved with each additional class. Classification quality as indicated by the entropy coefficient was high in Models 4–5. The relative improvement in fit with Model 4 was larger than that with Model 5 which largely replicated the patterns shown in Model 4 while identifying some classes with very small prevalence. We therefore selected the 4-class model as the most parsimonious description of the longitudinal patterns in the data. As can be seen in Fig. 1 which shows the four longitudinal types of maternal depressive symptoms across the four time-points, the most prevalent class (73.1%) included mothers with consistently low levels of symptoms throughout (‘consistently low’). At the other extreme were mothers with ‘chronically high’ depressive symptoms throughout, the lowest prevalence class (5.2%). The two intermediate classes included mothers with levels of symptoms that were moderate at the first two time-points but either increased (‘moderate-accelerating’) or decreased (‘moderate-decelerating’) with time (7.4 and 14.4%, respectively). As expected, maternal symptom class was related to family demographic and socio-economic factors, with the chronically high and the consistently low symptom groups being the most and the least disadvantaged, respectively. For example, mothers with chronically high levels of depressive symptoms were younger, poorer and less educated than the rest (results available on request).

**Table 2:** Log-likelihood and information criteria for alternative latent classes of maternal depressive symptoms

No. of classes	Log-likelihood	AIC	BIC	ssa BIC	Entropy
1	−117,405.972	234827.943	234,887.407	234,861.984	1
2	−109,986.197	219,998.395	220,095.024	220,053.711	0.925
3	−107,937.745	215,911.489	216,045.283	215,988.081	0.855
4	−106,952.249	213,950.498	214,121.457	214,048.365	0.874
5	−106,059.407	212,174.814	212,382.938	212,293.957	0.876
6	−105,181.063	210,428.126	210,673.415	210,568.545	0.860
7	−104,451.980	208,979.959	209,262.414	209,141.654	0.857

*AIC* Akaike information criterion, *BIC* Bayesian information criterion*, ssa BIC* sample-size-adjusted Bayesian information criterion

Next we explored sex differences and similarities in risky behaviours. As expected from theory and the descriptive analysis above, males scored higher than females in delinquency, antisocial behaviour, low moral judgement and positive attitudes to alcohol. However, there was no sex difference in perceived harm of alcohol use. The sex-stratified regression models subsequently fitted showed that maternal depressive symptom typology was associated with different outcomes in males and females^1^. As can be seen in Table 3 which shows the results for males, exposure to chronic or recently elevated maternal depressive symptoms predicted males’ antisocial behaviour. In females (Table 4), exposure to chronic maternal depressive symptoms was related to the view that alcohol use is harmless but also to high moral judgement. (Further analysis, however, showed that the effect on females’ moral judgment was likely due to suppression; chronic maternal depression was unrelated to females’ moral judgement in the unadjusted model and its regression coefficient became significant and changed direction when the covariates were added.) There were also notable sex differences (but important similarities, too) in the effects of the covariates. In both sexes, positive attitudes to alcohol were largely predicted by ethnicity, whereas antisocial behaviour and delinquency were largely predicted by socio-economic status and ethnicity and, especially in females, pubertal status. Low moral judgement and perceived harm of alcohol use were not predicted particularly well in either sex, although it seems that the former was related to poverty in both males and females. In females, it was also positively related to age and early puberty.

**Table 3:** Model estimates predicting delinquency, antisocial behaviour, low moral judgement and attitudes to alcohol in males

	Delinquency		Antisocial behaviour		Low moral judgement	
	Coeff. (SE)	95% CI	Coeff. (SE)	95% CI	Coeff. (SE)	95% CI
Constant	−0.589 (0.497)	[−1.566, 0.388]	0.607 (0.478)	[−0.333, 1.547]	−0.859 (0.535)	[−1.912, 0.193]
Maternal depressive symptom class (Ref. consistently low)						
Moderate-accelerating	0.123 (0.095)	[−0.064, 0.311]	0.197* (0.086)	[0.028, 0.367]	0.124 (0.107)	[−0.086, 0.333]
Moderate-decelerating	0.116 (0.075)	[−0.032, 0.263]	0.076 (0.062)	[−0.045, 0.198]	−0.022 (0.068)	[−0.155, 0.110]
Chronically high	0.083 (0.140)	[−0.193, 0.359]	0.350** (0.116)	[0.121, 0.578]	0.188 (0.113)	[−0.035, 0.410]
Age	0.035 (0.044)	[−0.053, 0.122]	−0.053 (0.043)	[−0.137, 0.032]	0.093 (0.048)	[−0.002, 0.188]
Puberty signs	0.068 (0.050)	[−0.030,c 0.166]	0.102* (0.044)	[0.015, 0.188]	0.032 (0.046)	[−0.059, 0.123]
Ethnicity (Ref. white)						
Mixed	−0.206* (0.090)	[−0.384, −0.029]	−0.195 (0.102)	[−0.395, 0.006]	0.061 (0.119)	[−0.173, 0.294]
Indian	−0.108 (0.086)	[−0.277, 0.062]	−0.274* (0.134)	[−0.539, −0.010]	−0.073 (0.137)	[−0.343, 0.196]
Pakistani/Bangladeshi	−0.406*** (0.108)	[−0.618, −0.195]	−0.546*** (0.117)	[−0.777, −0.315]	−0.118 (0.132)	[−0.379, 0.142]
Black	−0.362** (0.111)	[−0.581, −0.143]	−0.081 (0.138)	[−0.353, 0.191]	−0.113 (0.140)	[−0.388, 0.161]
Other	−0.378*** (0.089)	[−0.553, −0.202]	−0.581*** (0.116)	[−0.808, −0.353]	0.264 (0.651)	[−1.016, 1.544]
Mother’s age	0.001 (0.005)	[−0.008, 0.010]	−0.003 (0.004)	[−0.011, 0.005]	−0.001 (0.004)	[−0.010, 0.007]
Mother is university-educated	−0.071 (0.037)	[−0.143, 0.001]	0.014 (0.045)	[−0.075, 0.102]	−0.076 (0.047)	[−0.169 0.016]
No. of siblings	0.058* (0.028)	[0.003, 0.114]	0.080*** (0.021)	[0.038, 0.122]	0.003 (0.027)	[−0.050, 0.055]
No. of family status changes	0.130* (0.053)	[0.026, 0.235]	0.075 (0.043)	[−0.009, 0.160]	0.066 (0.048)	[−0.029, 0.161]
No. of sweeps in poverty	0.127*** (0.025)	[0.078, 0.176]	0.053** (0.020)	[0.013, 0.092]	0.062** (0.023)	[0.016, 0.108]

	Positive attitudes to alcohol		Low perceived harm of alcohol use	
	Coeff. (SE)	95% CI	Coeff. (SE)	95% CI
Constant	−0.735 (0.471)	[−1.662, 0.192]	−0.051 (0.457)	[−0.949, 0.848]
Maternal depressive symptom class (Ref. consistently low)				
Moderate-accelerating	0.047 (0.080)	[−0.110, 0.203]	−0.032 (0.090)	[−0.208, 0.144]
Moderate-decelerating	−0.077 (0.058)	[−0.190, 0.036]	0.087 (0.058)	[−0.028, 0.202]
Chronically high	−0.035 (0.114)	[−0.259, 0.188]	0.098 (0.113)	[−0.125, 0.320]
Age	0.076 (0.042)	[−0.007, 0.159]	0.010 (0.043)	[−0.074, 0.095]
Puberty signs	0.052 (0.037)	[−0.021, 0.126]	−0.040 (0.043)	[−0.124, 0.044]
Ethnicity (Ref. white)				
Mixed	−0.138 (0.104)	[−0.341, 0.066]	−0.136 (0.115)	[−0.363, 0.091]
Indian	−0.436** (0.153)	[−0.737, −0.135]	−0.291 (0.161)	[−0.608, 0.027]
Pakistani/Bangladeshi	−0.687*** (0.088)	[−0.859, −0.515]	0.055 (0.141)	[−0.223, 0.333]
Black	−0.270* (0.124)	[−0.514, −0.027]	−0.202 (0.114)	[−0.426, 0.022]
Other	−0.801*** (0.140)	[−1.076, −0.526]	0.218 (0.250)	[−0.273, 0.708]
Mother’s age	0.004 (0.004)	[−0.003, 0.011]	−0.001 (0.004)	[−0.007, 0.006]
Mother is university-educated	0.014 (0.043)	[−0.071, 0.100]	−0.030 (0.041)	[−0.111, 0.050]
No. of siblings	−0.018 (0.022)	[−0.061, 0.025]	0.014 (0.020)	[−0.024, 0.053]
No. of family status changes	−0.000 (0.035)	[−0.070, 0.070]	0.002 (0.039)	[−0.075, 0.079]
No. of sweeps in poverty	0.008 (0.019)	[−0.029, 0.045]	−0.012 (0.019)	[−0.049, 0.026]

*p* < 0.05; ** p < 0.01; *** p < 0.001

**Table 4:** Model estimates predicting delinquency, antisocial behaviour, low moral judgement and attitudes to alcohol in females

	Delinquency		Antisocial behaviour		Low moral judgement	
	Coeff. (SE)	95% CI	Coeff. (SE)	95% CI	Coeff. (SE)	95% CI
Constant	−1.120** (0.375)	[−1.856, −0.383]	−0.689 (0.359)	[−1.395, 0.017]	−1.012** (0.338)	[−1.677, −0.348]
Maternal depressive symptom class (Ref. consistently low)						
Moderate-accelerating	0.052 (0.062)	[−0.070, 0.174]	−0.007 (0.068)	[−0.141, 0.127]	0.037 (0.077)	[−0.115, 0.189]
Moderate-decelerating	0.036 (0.042)	[−0.047, 0.119]	0.092 (0.053)	[−0.012, 0.196]	0.077 (0.055)	[−0.030, 0.184]
Chronically high	0.075 (0.070)	[−0.062, 0.211]	0.056 (0.090)	[−0.120, 0.233]	−0.138* (0.060)	[−0.256, −0.020]
Age	0.070* (0.034)	[0.002, 0.137]	0.034 (0.035)	[−0.034, 0.102]	0.078* (0.031)	[0.018, 0.138]
Puberty signs	0.072* (0.029)	[0.015, 0.129]	0.111* (0.045)	[0.023, 0.199]	0.116** (0.037)	[0.043, 0.190]
Ethnicity (Ref. white)						
Mixed	0.030 (0.092)	[−0.151, 0.211]	−0.078 (0.097)	[−0.269, 0.114]	0.189 (0.143)	[−0.091, 0.470]
Indian	−0.034 (0.071)	[−0.173, 0.106]	−0.108 (0.092)	[−0.289, 0.072]	−0.142* (0.058)	[−0.255, −0.028]
Pakistani/Bangladeshi	−0.064 (0.121)	[−0.302, 0.175]	−0.285** (0.091)	[−0.464, −0.106]	−0.146 (0.082)	[−0.307, 0.016]
Black	−0.160** (0.059)	[−0.277, −0.043]	0.057 (0.075)	[−0.092, 0.205]	−0.062 (0.087)	[−0.232, 0.109]
Other	−0.202*** (0.034)	[−0.268, −0.135]	−0.291** (0.109)	[−0.506, −0.076]	0.025 (0.085)	[−0.142, 0.192]
Mother’s age	0.005 (0.003)	[−0.001, 0.010]	0.000 (0.003)	[−0.006, 0.005]	−0.003 (0.003)	[−0.009, 0.003]
Mother is university-educated	−0.069** (0.023)	[−0.114, −0.024]	−0.015 (0.033)	[−0.079, 0.049]	−0.068 (0.038)	[−0.142, 0.006]
No. of siblings	−0.001 (0.016)	[−0.032, 0.031]	0.031 (0.018)	[−0.003, 0.065]	0.012 (0.021)	[−0.029, 0.052]
No. of family status changes	0.039 (0.033)	[−0.027, 0.104]	0.038 (0.032)	[−0.024, 0.101]	0.044 (0.033)	[−0.021, 0.109]
No. of sweeps in poverty	0.081*** (0.016)	[0.050, 0.111]	0.058** (0.017)	[0.025, 0.092]	0.031* (0.016)	[0.000, 0.062]

	Positive attitudes to alcohol		Low perceived harm of alcohol use	
	Coeff. (SE)	95% CI	Coeff. (SE)	95% CI
Constant	−1.474** (0.430)	[−2.319, −0.629]	0.717 (0.431)	[−0.129, 1.564]
Maternal depressive symptom class (Ref. consistently low)				
Moderate-accelerating	0.106 (0.076)	[−0.043, 0.255]	−0.008 (0.077)	[−0.159, 0.142]
Moderate-decelerating	0.088 (0.051)	[−0.011, 0.188]	0.063 (0.043)	[−0.022, 0.148]
Chronically high	0.001 (0.096)	[−0.187, 0.189]	0.249* (0.114)	[0.024, 0.474]
Age	0.101* (0.039)	[0.024, 0.178]	−0.065 (0.038)	[−0.139, 0.010]
Puberty signs	0.065 (0.046)	[−0.026, 0.156]	−0.049 (0.057)	[−0.161, 0.063]
Ethnicity (Ref. white)				
Mixed	−0.147 (0.099)	[−0.342, 0.047]	−0.317*** (0.087)	[−0.488, −0.146]
Indian	−0.274* (0.108)	[−0.487, −0.061]	−0.043 (0.164)	[−0.364, 0.279]
Pakistani/Bangladeshi	−0.538*** (0.095)	[−0.726, −0.351]	0.027 (0.118)	[−0.205, 0.259]
Black	−0.365** (0.134)	[−0.628, −0.103]	−0.137 (0.198)	[−0.527, 0.252]
Other	−0.321 (0.204)	[−0.722, 0.081]	−0.162 (0.205)	[−0.565, 0.240]
Mother’s age	0.007* (0.003)	[0.001, 0.014]	0.002 (0.004)	[−0.005,0.009]
Mother is university-educated	0.003 (0.041)	[−0.077, 0.084]	−0.038 (0.039)	[−0.114, 0.038]
No. of siblings	0.009 (0.018)	[−0.026, 0.045]	−0.021 (0.019)	[−0.059, 0.017]
No. of family status changes	0.044 (0.033)	[−0.020, 0.108]	0.008 (0.036)	[−0.062, 0.078]
No. of sweeps in poverty	0.010 (0.016)	[−0.022, 0.041]	0.030 (0.020)	[−0.009, 0.069]

*p* < 0.05; ** p < 0.01; *** p < 0.001

## Discussion

This study examined the effect of trajectories of maternal depressive symptoms in childhood on risky behaviours and some of their associated attitudes at the end of primary school (age 11 years). To our knowledge, this is the first study to examine this with children at the cusp of adolescence, when engagement in risky behaviours is likely to set the stage for a range of poor outcomes later on [23–25]. Using data from a large population-based sample in the UK and adjusting for important covariates of both maternal depression and youth risky behaviours, it found, as expected, that the longitudinal typology of maternal depressive symptoms in childhood predicted some of these attitudes and behaviours, although differently by sex. In females, exposure to chronically high maternal depressive symptoms was associated with the view that alcohol use is harmless. In males, exposure to chronically high or recently elevated maternal depressive symptoms was related to loud and rowdy behaviour, alcohol use and bullying. These are important findings because some of these behaviours were not uncommon in our sample. For example, over a third of the males in our sample had bullied peers and almost a quarter reported that they had been loud or rowdy in public. Thus, preventing or treating high maternal depressive symptoms in childhood may be an effective strategy for reducing a range of common risky behaviours in early adolescent males. It may also be useful in reducing alcohol use in early adolescent females. Perceived harm of alcohol use has been shown to predict, directly or indirectly, alcohol use [4] and so attitudes about alcohol could serve as screeners and intervention targets for adolescents who may be considering starting to use it^2^ In early adolescence, therefore, shifts towards more positive attitudes to alcohol use could alert parents, physicians and teachers to potential alcohol use initiation [30].

Why would chronic depressive symptoms in mothers predict risky behaviours only in male adolescents? The overall pattern of greater risk from maternal depressive symptoms for boys is consistent with previous findings [27]. As for the reason why maternal depressive symptoms would predict youth risky behaviours in the first place, ongoing maternal symptoms may be a marker of genetic risk [26] but maternal depressive symptoms also co-occur with family stress and less engaged and supportive parenting [19], in turn predicting externalising problems and health-risk behaviours [20, 21]. Another (related) possibility is that the effects of maternal depressive symptoms found may simply be proxies for the influences of paternal antisocial behaviour [11] and family attitudes to alcohol [12], neither of which was measured in MCS. We think, however, that this weakness is outweighed by the study’s many strengths. These include the use of data from a large and nationally representative cohort, the longitudinal design, the prospective collection of maternal depressive symptoms and, as discussed above, the examination of the role of maternal depressive symptoms in a range of risky behaviours and associated attitudes in very early adolescence.

Regardless, our findings must be seen in context. First, our measure of depressive symptoms, the Kessler K6, is a measure of psychological distress rather than depression. However, it has been found to have an estimated area under the curve of around 0.90 against a standard diagnostic assessment of depression (the World Mental Health Composite International Diagnostic Interview module for major depression) [29]. Second, effects were weak. Much previous research has established that maternal depression is related to parent–child conflict over autonomy and control, increased peer pressures and less parental monitoring and supervision [19], in turn linked with externalising behaviour and risk-taking in adolescence [20, 21]. However, maternal depressive symptomatology was neither the most consistent nor the most powerful predictor of the youth outcomes examined in this study. For both sexes, the important predictors of risky behaviours appeared to be, instead, white ethnicity and low socio-economic status (and early puberty, especially for females). Nonetheless, our study suggests that, if the associations found are causal, preventing or treating maternal depression may reduce antisocial behaviour in early adolescent males and alcohol use in adolescent females.

## Footnotes

1 Our decision to fit sex-stratified models was supported empirically, too. There were significant interactions between maternal depressive symptom typology and sex on antisocial behaviour, low perceived harm of alcohol use, and positive attitudes to alcohol. The interactions between the two were marginally significant for low moral judgement and delinquency.

2 We note, however, that in our sample of 11 year olds the concurrent correlation between attitudes to alcohol and self-reported alcohol use was significant but weak (around 0.15), as were the concurrent correlations between attitudes to alcohol and frequency or quantity of drinking among those who reported that they had tried alcohol (never exceeding 0.19). At the time of writing we did not have access to MCS6 (age 14 sweep) to explore if attitudes to alcohol at age 11 years predict alcohol use at age 14 years.

## References

[1] Wickham ME, Senthilselvan A, Wild TC, Hoglund WL, Colman I (2015). Maternal depressive symptoms during childhood and risky adolescent health behaviors. Pediatrics.

[2] Radliff KM, Wheaton JE, Robinson K, Morris J (2012). Illuminating the relationship between bullying and substance use among middle and high school youth. Addict Behav.

[3] Stams GJ, Brugman D, Deković M, van Rosmalen L, van der Laan P, Gibbs JC (2006). The moral judgment of juvenile delinquents: a meta-analysis. J Abnorm Child Psychol.

[4] Patrick ME, Schulenberg JE (2010). Alcohol use and heavy episodic drinking prevalence and predictors among national samples of American eighth- and tenth-grade students. J Stud Alcohol Drugs.

[5] Barker ED, Copeland W, Maughan B, Jaffee SR, Uher R (2012). Relative impact of maternal depression and associated risk factors on offspring psychopathology. Br J Psychiatry.

[6] Kretschmer T, Oliver BR, Maughan B (2014). Pubertal development, spare time activities, and adolescent delinquency: testing the contextual amplification hypothesis. J Youth Adolesc.

[7] Kessler RC, Barker PR, Colpe LJ, Epstein JF, Gfroerer JC, Hiripi E (2003). Screening for serious mental illness in the general population. Arch Gen Psychiatry.

[8] Mahalik JR, Levine Coley R, McPherran Lombardi C, Doyle Lynch A, Markowitz AJ, Jaffee SR (2013). Changes in health risk behaviors for males and females from early adolescence through early adulthood. Health Psychol.

[9] Moss HB, Chen CM, Yi HY (2014). Early adolescent patterns of alcohol, cigarettes, and marijuana polysubstance use and young adult substance use outcomes in a nationally representative sample. Drug Alcohol Depend.

[10] DeLisi M, Neppl TK, Lohman BJ, Vaughn MG, Shook JJ (2013). Early starters: which type of criminal onset matters most for delinquent careers?. J Crim Just.

[11] Marmorstein NR, Malone SM, Iacono WG (2004). Psychiatric disorders among offspring of depressed mothers: associations with paternal psychopathology. Am J Psychiatry.

[12] Kelley ML, Lawrence HR, Milletich RJ, Hollis BF, Henson JM (2015). Modeling risk for child abuse and harsh parenting in families with depressed and substance-abusing parents. Child Abuse Negl.

[13] Hammen C, Brennan PA (2003). Severity, chronicity, and timing of maternal depression and risk for adolescent offspring diagnoses in a community sample. Arch Gen Psychiatry.

[14] Pawlby S, Hay DF, Sharp D, Waters CS, O’Keane V (2009). Antenatal depression predicts depression in adolescent offspring: prospective longitudinal community-based study. J Affect Disord.

[15] Hay DF, Pawlby S, Waters CS, Sharp D (2008). Antepartum and postpartum exposure to maternal depression: different effects on different adolescent outcomes. J Child Psychol Psychiatry.

[16] Campbell SB, Morgan-Lopez AA, Cox MJ, McLoyd VC (2009). A latent class analysis of maternal depressive symptoms over 12 years and offspring adjustment in adolescence. J Abnorm Psychol.

[17] Hammerton G, Mahedy L, Mars B, Harold GT, Thapar A, Zammit S, Collishaw S (2015). Association between maternal depression symptoms across the first 11 years of their child’s life and subsequent offspring suicidal ideation. PLoS One.

[18] Hallfors DD, Waller MW, Bauer D, Ford CA, Halpern CT (2005). Which comes first in adolescence: sex and drugs or depression?. Am J Prev Med.

[19] Lovejoy MC, Graczyk PA, O’Hare E, Neuman G (2000). Maternal depression and parenting behavior: a meta-analytic review. Clin Psychol Rev.

[20] Bahr SJ, Hoffmann JP, Yang X (2005). Parental and peer influences on the risk of adolescent drug use. J Prim Prev.

[21] Barnes GM, Hoffman JH, Welte JW, Farrell MP, Dintcheff BA (2006). Effects of parental monitoring and peer deviance on substance use and delinquency. J Marriage Fam.

[22] Farrell AD, Sullivan TN, Esposito LE, Meyer AL, Valois RF (2005). A latent growth curve analysis of the structure of aggression, drug use, and delinquent behaviors and their interrelations over time in urban and rural adolescents. J Res Adolesc.

[23] Hingson RW, Heeren T, Winter MR (2006). Age at drinking onset and alcohol dependence: age at onset, duration, and severity. Arch Pediatr Adolesc Med.

[24] Jackson KM, Barnett NP, Colby SM, Rogers ML (2015). The prospective association between sipping alcohol by the sixth grade and later substance use. J Stud Alcohol Drugs.

[25] Piquero AR, Chung HL (2000). On the relationships between gender, early onset, and the seriousness of offending. J Crim Justice.

[26] Kim-Cohen J, Moffitt TE, Taylor A, Pawlby SJ, Caspi A (2005). Maternal depression and children’s antisocial behavior: nature and nurture effects. Arch Gen Psychiatry.

[27] Connell AM, Goodman SH (2002). The association between psychopathology in fathers versus mothers and children’s internalizing and externalizing behavior problems: a meta-analysis. Psychol Bull.

[28] Ellis BJ, Garber J (2000). Psychosocial antecedents of variation in girls’ pubertal timing: maternal depression, stepfather presence, and marital and family stress. Child Dev.

[29] Cairney J, Veldhuizen S, Wade TJ (2007). Evaluation of 2 measures of psychological distress as screeners for depression in the general population. Can J Psychiatry.

[30] Maggs JL, Staff J, Patrick ME, Wray-Lake L, Schulenberg JE (2015). Alcohol use at the cusp of adolescence: a prospective national birth cohort study of prevalence and risk factors. J Adolesc Health.

